# From Collection or Archaeological Finds? A Non-Destructive Analytical Approach to Distinguish between Two Sets of Bronze Coins of the Roman Empire

**DOI:** 10.3390/molecules28052382

**Published:** 2023-03-05

**Authors:** Giovanna Marussi, Matteo Crosera, Enrico Prenesti, Bruno Callegher, Elena Baracchini, Gianluca Turco, Gianpiero Adami

**Affiliations:** 1 Dipartimento di Scienze Chimiche e Farmaceutiche, Università degli Studi di Trieste, 34127 Trieste, Italy; 2 Dipartimento Interateneo di Scienze, Progetto e Politiche del Territorio, Università degli Studi di Torino, 10125 Torino, Italy; 3 Dipartimento di Studi Umanistici, Università degli Studi di Trieste, 34124 Trieste, Italy; 4 Dipartimento Universitario Clinico di Scienze Mediche Chirurgiche e della Salute, Università degli Studi di Trieste, 34125 Trieste, Italy

**Keywords:** Roman coins, numismatics, metals, alloys, bronze, patinas, micro-EDXRF, ICP-AES, FTIR-ATR, SEM-EDS

## Abstract

This study stems from the need for numismatics to establish whether there may be relationships between a group of 103 bronze coins from the Roman era found in archaeological excavations on the Cesén Mountain (Treviso, Italy) and a group of 117 coins kept at the Museum of Natural History and Archaeology in Montebelluna (Treviso, Italy). The chemists were delivered six coins with neither pre-agreements nor further information on the origin of the coins. Therefore, the request was to hypothetically assign the coins to the two groups on the basis of similarities and differences in their surface composition. Only non-destructive analytical techniques were allowed to be used to characterize the surface of the six coins taken blindly from the two sets. The elemental analysis of each coins’ surface was carried out by µ-XRF. To better observe the morphology of the coins’ surfaces, SEM-EDS was used. Compounds covering the coins coming from both corrosion processes (patinas) and the deposition of soil encrustations were also analyzed by means of the FTIR-ATR technique. The molecular analysis confirmed the presence of silico-aluminate minerals on some coins, unequivocally indicating a provenance from clayey soil. Some soil samples, collected from the archaeological site of interest, were analyzed to verify whether the encrusted layer on coins could contain chemical components compatible with them. This result, together with the chemical and morphological investigations, led us to subdivide the six target coins into two groups. The first group is made up of two coins coming from the set of coins from excavation (found in the subsoil) and from the set from open air finds (coins found in the top layer of the soil). The second group is made up of four coins that are devoid of characteristics corresponding to exposure to soil contact for long periods of time and, moreover, their surface compounds could suggest a different provenance. The analytical results of this study made it possible to correctly assign all six coins to the two groups of finds and support numismatics, which was unconvinced in considering all coins to come from the same finding site only on the basis of archaeological documentations.

## 1. Introduction

Metal artefacts and, in particular, ancient coins are very important in order to obtain more information about history, the evolution of mankind, and the economy of the minting period. The critical point is that they are very sensitive to the effect of deterioration processes; in fact, their state of preservation depends on pre-burial wear and on all of the chemical and physical alterations in the burial environment. Several environmental factors contribute to the modification of the chemical composition of ancient metal artefacts, such as pH; the presence of oxygen and marine, urban, or agricultural corrosive agents; temperature cycles; and humidity [1,2]. 

The metallographic characteristics of ancient bronze artefacts often conceal peculiar and specific corrosion behaviour, which can provide valuable tools and information to conservators and archaeologists [3]. As a result of burial and corrosion, on the surface of artefacts, especially copper-based artefacts, several insoluble compounds can form, usually as successive, irregular, and disintegrated layers. From the analysis of these, it is possible to trace the burial site of the samples. Various works have focused on the study of bronze artefacts and their burial environments, such as some Cu-based archaeological artefacts found during archaeological excavations in Turkey, Jordan, and Italy [4] and Montefortino helmets found in the Mediterranean seabed [5]. Among the most widely used analytical techniques for this purpose is X-ray fluorescence (XRF), which allows a semi-quantitative elemental analysis of the surface of samples [6,7].

With reference to this case study, the elemental composition of an archaeological patina of ancient bronze coins found in the subsoil is usually very complex and results from the deterioration processes, generically named corrosion, that occur during centuries of burial.

The corrosion products covering the coins are strictly correlated to the environmental characteristics of the recovery site [2,8]. Thus, the chemical analysis of the coins’ surfaces can be a useful tool for numismatists to obtain clues and information on the history of finds [9]. A different surface composition of materials that are similar in bulk, in fact, could likely be due to different micro-environmental conditions of the archeological site of recovery [2,3,4,5,6,7,8,10].

This type of research is inscribed in the line of studies on bronze artifacts of archaeological origin aimed at determining the chemical characteristics and structure of the patinas formed on them following their existence for centuries in corrosive environments [11].

**Figure 1:**
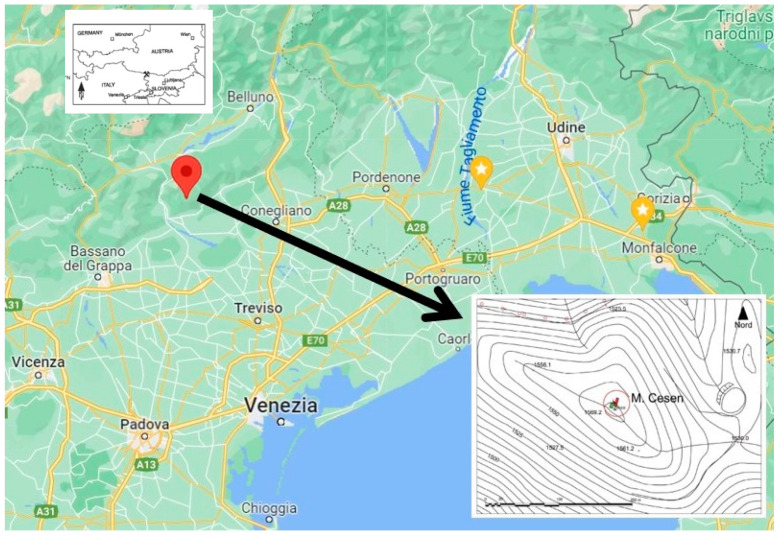
Location of Cesén Mountain, the survey site.

In 2012, an excavation led to the discovery of a possible place of worship visited by migrant people on the pass of the Cesén Mountain, a massif of the Venetian Pre-Alps located at the north-western edge of the province of Treviso, Italy (see the map in Figure 1); the highest peak is 1570 m AMSL [12]. In contrast to what normally happens in archeological sites, the ceramic findings were small in amount, having probably been destroyed by harsh winters; nevertheless, the numismatic finds are noteworthy and deserve attention for the purpose of obtaining information. The archaeological area under study has similarities with other sanctuaries found in Venetia et Histria (north-eastern Italy). The findings of bronze coins dated from the first to the fourth centuries AD suggest the discovery of a possible votive deposit visited by transhumant shepherds, located on the transhumance paths from the alpine region to plane pasturelands.

To outline the hypothesis of the votive character of the place, there is also the discovery of some blocks of limestone connected to the collapse of a low wall, which, in all probability, delimited the cult area where the coins were laid down in small dimples covered with scales of biancone (a local name to indicate a limestone lithological formation). The coins were found in multiple cavities around the cult area.

This study stems from the need for numismatics to establish whether there may be relationships between (i) bronze coins (117 specimens that date back between the first and fourth centuries AD) kept at the Museum of Natural History and Archaeology in the municipality of Montebelluna (Treviso, Italy), found during various archeological surveys on Cesén Mountain before 2012 (the first discovery dates back to 1996), and (ii) bronze coins (103 specimens from the Roman imperial era) found in 2012 during some archaeological excavations on Cesén Mountain [12].

**Figure 2:**
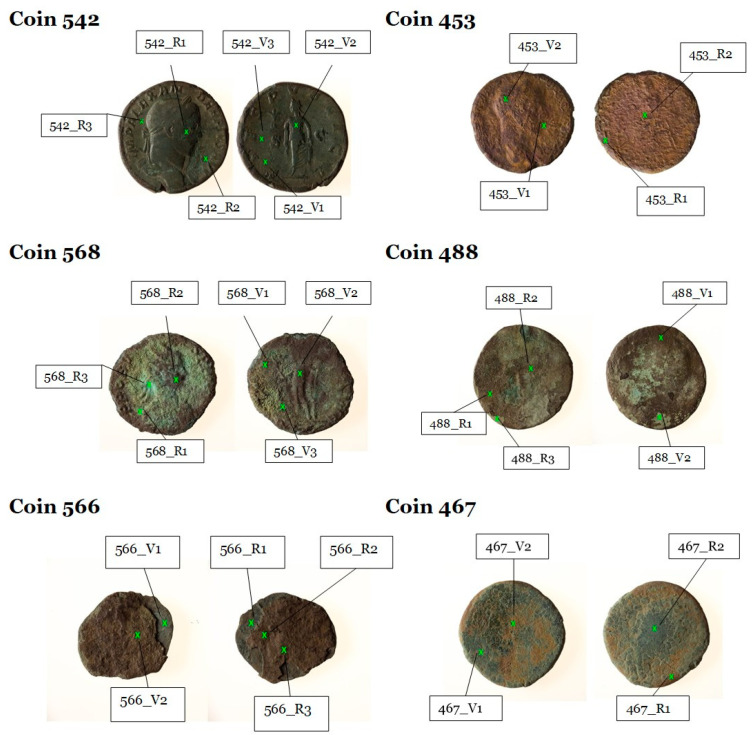
Sampling points for the six target coins analyzed by µ-XRF (R = recto; V = verso).

Six target coins were blindly taken from the two sets of specimens in order to highlight similarities and differences between the groups. The chemists were delivered six coins (see Figure 2) with neither pre-agreements nor further information; the task was to identify if it was a compositionally homogeneous set or if the coins were attributable to two different groups of finds. The limited availability of coins’ specimens usable for the analyses is a constraint established by the institutional body who commissioned the study [13,14,15]. As the analysts did not know the size of the two groups of coins until the end of the study, six coins were considered sufficient to test the method. In fact, the probability of correctly assigning the six coins to the two groups of finds in a random manner is 1.56%.

To avoid damage to the numismatic finds, only non-destructive analytical techniques were allowed to be used in order to characterize the surface of the coins [16,17]. The elemental analysis of each coins’ surface was carried out by µ-X-ray fluorescence spectroscopy (µ-XRF) [18]. To better observe the morphology of the coins’ surfaces, scanning electron microscopy coupled to energy dispersive spectroscopy (SEM-EDS) was used [19].

The µ-XRF technique was first employed to identify the presence of elements other than the major element, copper, which is often used as the reference element [20,21]. 

This technique is useful to gather information about the surface composition of a material, as only low depths can be reached by the analytical ray. On the other hand, no treatment of the sample was required.

Compounds covering the coins coming from both corrosion processes (patinas) and depositions of soil encrustations were also analyzed by means of the FTIR-ATR (Fourier transform infrared spectroscopy–attenuated total reflection) technique.

To strengthen the investigation, three soil samples, collected from the archaeological site of interest, were analyzed to verify whether the encrusted layer of coins could contain chemical components compatible with the elemental composition of the soil. 

It is important to point out that, until the end of the study, analysts had no information regarding the origin of the coins, the issue period, or the composition, nor was any reference to the historical-numismatic hypotheses made.

## 2. Results and Discussion

**Figure 3:**
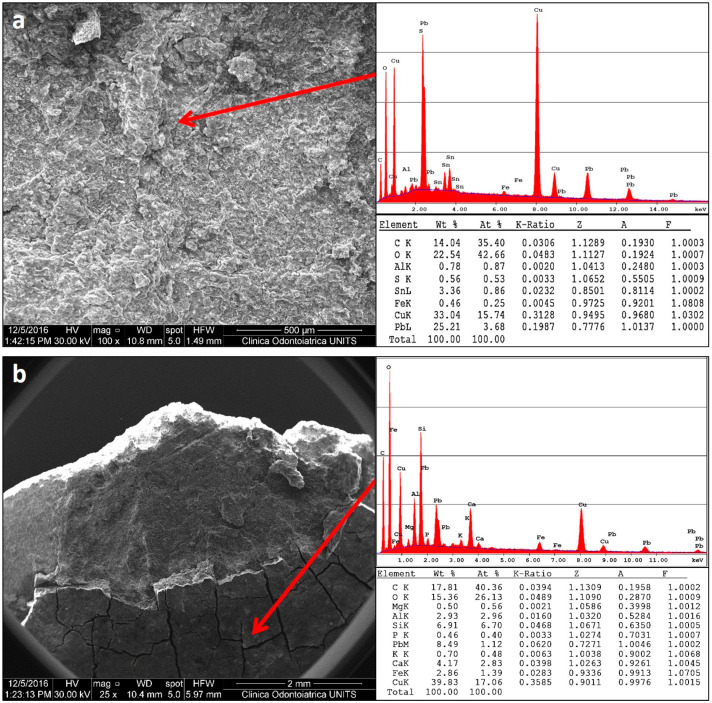
(**a**,**b**): Representative SEM micrographs and relative EDX spectra with standardless quantification (element normalized) of coin 566: (**a**) bulk and (**b**) soil concretions.

The SEM-EDX investigation was carried out on three coins, namely, 488, 542, and 566. This preliminary analysis was useful to observe the morphology of the coins’ surfaces. In Figure 3a, the results for coin 566, obtained on a clean area, show the presence of copper (about 33%) and lead (about 25%) in the bulk of the coin. Figure 3b shows the SEM-EDX spectrum on the area covered by the soil concretions, which consist essentially of Ca (4%), Si (about 7%), Al (about 3%), and Fe (about 3%), while Pb % decreases a lot and Cu % remains at previous levels of about 35. 

**Figure 4:**
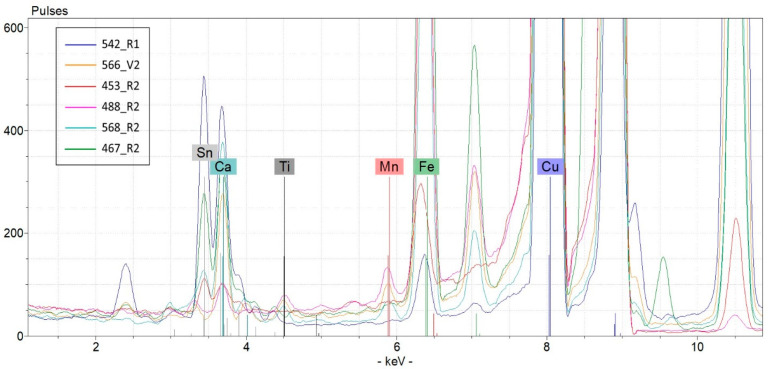
Representative µ-XRF spectra of the six coins.

µ-XRF measurements were performed on both the clean and altered (corroded or encrusted) areas and at least two spectra were collected on the recto and two on the verso of each coin in order to obtain representative data of the entire specimen (see Figure 4). 

The signal counts of the elements of interest are shown in Table 1. By calculating the area subtended by the peaks relative to the copper Kα1 line and to the lead Lα1 line and tin Kα2 line, it was possible to calculate the ratios Pb/Cu and Sn/Cu in order to delineate the trend of these metals at the surface (see Table 1).

To obtain more interpretable data of the element surface composition of the six coins, for each spectrum, the percentage ratio of the peak intensities (expressed in counts) of each element to the sum of the peak intensities of all the elements considered and measurable by the µ-XRF spectrum was calculated. The values obtained for the different points of the same coin were averaged (see Table 2); a high coefficient of variation expressed as a percentage relative standard deviation indicates a non-homogeneous distribution of the elements on the surface of the coin. This may be indicative of a different environmental history for the coins. 

On the basis of these µ-XRF data, we can affirm the following:

1. coin 542 differs from the others owing to its lower Cu content and highest content of Pb and Sn;
2. coin 467 contains Zn, an element that is under the limit of detection in the other specimens; Roman bronze was a typical alloy made of Cu, Sn, Pb, and Zn, and the Romans manufactured copper alloys by combining variable percentages of alloys containing Sn and Zn; the result was an alloy with a composition ranging between that of common bronzes and brass;
3. coin 568 contains Ag, an element that is under the limit of detection in the other specimens;
4. coins 568 and 566 are related by the presence of Ti and Mn, which are typical elements of the ground;
5. coins 453 and 488 are similar to each other in terms of their Cu content, which, for both coins, is close to the totality.

These results indicate a wide chemical heterogeneity of these finds—even within the substantial common tin bronze alloy identity—revealing different histories. This outcome allows to sustain the hypothesis that the coins under study were found in a place visited by migrant people on Cesén Mountain.

Conventional bronze alloy is usually based on Cu and Sn as the main alloy elements, even though arsenical bronze (probably the first alloy of the wide bronze family) and Roman bronze are known to have Zn. We found some unexpected elements in various combinations, often fortuitous and mirroring the variability of raw materials. Ti and Mn were found only on the surface of two coins (namely 566 and 568). In particular, Ti, Mn, Fe, and Ca are typical terrigenous elements and were in fact also found in the soil sampled in this archeological site (see Table 3). The surface of coins 566 and 568 bears a covering of brown earthy crust, indicating a discovery of the finds in the subsoil, probably after being underground for a long time. Specifically, regarding coin 488, the Ti signal is present only in the µ-XRF spectrum acquired in point 488_R1. This fact can be considered as a simple fortuitous contamination, and it sharply discourages the inclusion of this coin in the small group of specimens found in the subsoil.

**Figure 5:**
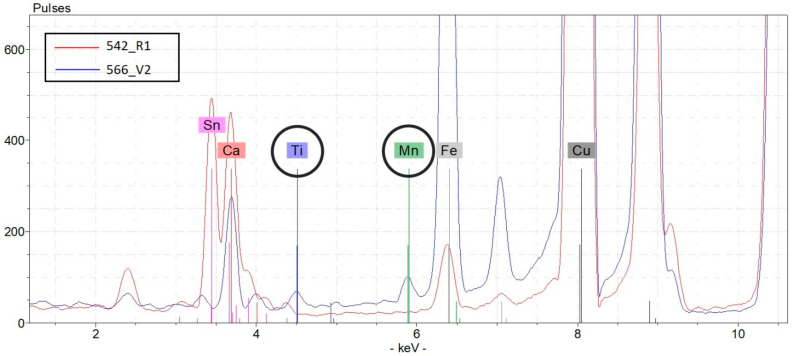
µ-XRF spectra comparison between a representative point (566_V2) of the superficial patina of coin 566 and a point (542_R1) of coin 542.

For the sake of example, Figure 5 shows the comparison between the µ-XRF spectrum related to a representative point (566_V2) of the superficial patina of coin 566 and a point (542_R1) of coin 542 that shows the presence of Ti and Mn signals in the first case and their absence in the second case. The same comparison can be made between the spectra of the most indicative points of all of the coins.

**Figure 6:**
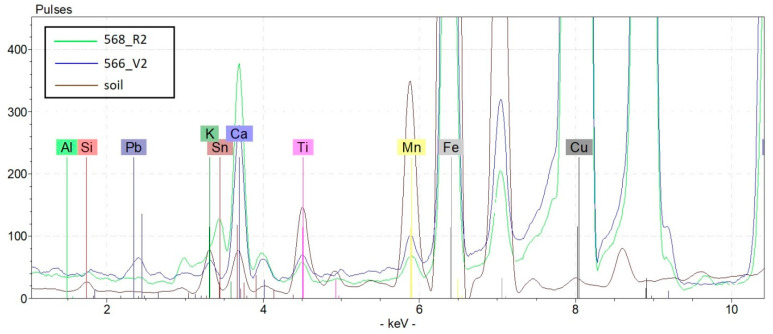
µ-XRF spectra comparison between points 566_V2 (blue line), 568_R2 (green line), and the soil sample (brown).

From the µ-XRF spectra of soil samples (Table 1) coming from the excavation site (see also the quantitative concentration results collected in Table 3), one can note that (Figure 6), limited to the presence of Ti and Mn, only the profiles relating to coins 566 and 568 seem to be compatible with that of the local soil.

A fairly good correlation (correlation coefficient > 0.7) between Ti and Mn signal peaks was found, thus fortifying the hypothesis of soil attribution of these exogenous elements as to those conventionally foreseeable for a common bronze (even beyond any minor metals present in the raw materials). The µ-XRF signal ratio Ti/Mn was 0.62 for soils and has a very similar value of 0.60 for coin 568 (average of spectra 568_R1, 568_R2, and 568_V2).

FTIR-ATR analyses on coins 488 and 566, carried out in both cleaned areas and encrusted areas, confirmed the presence of silico-aluminate minerals, unequivocally indicating a provenance from clayey soil (at least to the depth of the find).

**Figure 7:**
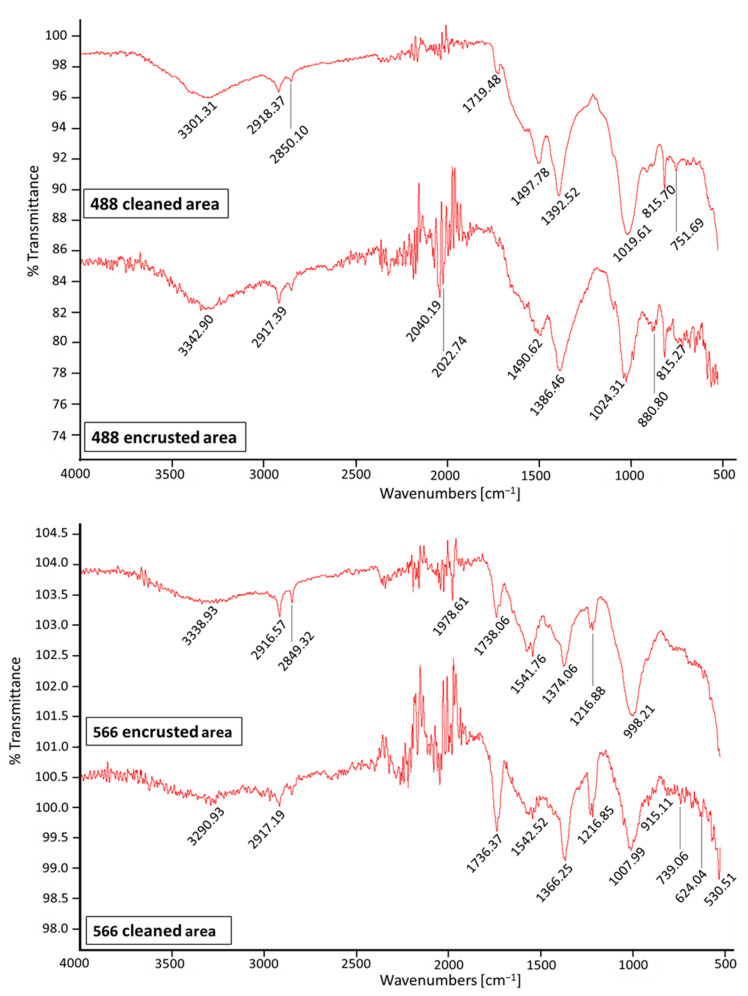
FTIR-ATR spectra of coins 488 and 566, carried out in both cleaned and encrusted areas.

Carbonates (calcareous soils) show (see Figure 7) strong absorption bands near 1400 and 870 cm^−1^, and we can observe a peak for coin 566 at 1392 cm^−1^ due to calcite and/or dolomite minerals [22].

For both coins, we can observe the presence of the typical peak of kaolinite (Al_2_(OH)_4_Si_2_O_5_) in the range of 1000–1100 cm^−1^ due to asymmetric stretching of silicates. Silico-aluminates (clayey soils) also show IR absorption bands around 630, 800, and 850 cm^−1^ [11,22,23,24,25].

Sulphates (CaSO_4_ and related hydrated salts) are not detectable in all IR spectra; the characteristic corrugated absorption bands in the range of 3600–3300 cm^−1^ and a typical small band at about 1620 cm^−1^ are in fact absent.

## 3. Materials and Methods

### 3.1. Chemicals and Apparatuses

All chemicals used were of analytical grade. Nitric acid (≥69% *w*/*w*), hydrochloric acid (≥37% *w*/*w*), hydrofluoric acid (48%), boric acid (99.99%), and hydrogen peroxide (30%) were purchased from Sigma Aldrich (Milan, Italy). Water of reagent grade was produced with a Millipore purification pack system (MilliQ water). Standards of Ca, Fe, Al, Mg, K, Ti, and Mn used for the calibration curves related to ICP-AES analyses were obtained by dilution (in 2.5% HNO_3_ aqueous solution) of SPECTRASCAN^®^ 1000 mg/L standard solutions purchased from Teknolab (Drøbak, Norway). 

### 3.2. Elemental Analyses

#### 3.2.1. Sample Acquisition

##### Coins

As the study was carried out blindly, information on the group to which each coin belongs was disclosed only at the end of the investigations. Both groups of specimens are declared as being found in the same area during archaeological excavations carried out at different times. 

The six specimens of coins provided for analyses were identified with a general inventory (GI) number (marked “IG” in Italian language with the same meaning) as follows:

IG369542 = appendix n. 57: coin #542

IG369568 = catalogue n. 102: coin #568

IG369566 = catalogue n. 103: coin#566

IG369453 = appendix n. 59: coin #453

IG369488 = appendix n. 20: coin #488

IG369467 = appendix n. 102: coin #467 

The term “appendix” indicates the coins from recent archaeological surveys, while the term “catalogue” indicates the coins from the quoted museum collection. 

The chemical analyses were performed at least on two points per coin on both sides. The measurement points are evidenced in pictures shown in Figure 2 (where R = recto and V = verso). 

##### Soils

Soil samples were collected by means of a plastic spoon and a plastic bag in the archaeological site of the find, and then dried for two days before undergoing chemical analysis. Soil samples were analyzed according to two techniques: (i) without pre-treatment by means of µ-XRF (a non-destructive technique) and (ii) after acid digestion, by means of destructive inductively coupled plasma–atomic emission spectroscopy (ICP-AES). 

#### 3.2.2. µ-XRF Qualitative Measurements

The elemental analysis of each coin’s surface was carried out by micro-energy dispersive X-ray fluorescence spectrometry (µ-EDXRF, briefly µ-XRF). An ARTAX 200 µ-XRF spectrometer (supplied by Bruker Nano GmbH) was used. The instrument was setup with the following test parameters: X-ray tube, 30 W, Mo target U = 50 kV, I = 700 μA, acquisition time: 60 s (live time), and collimator: 650 μm (air environment). The instrument consists of an air-cooled Mo X-ray fine focus tube (max 50 kV, 1 mA, 40 W) controlled by a compact high voltage generator unit and equipped with a 650 mm collimator. It is equipped with a Peltier cooled XFlash^®^ silicon drift detector (10 mm^2^ of active area and energy resolution <150 eV for Mn–Ka at 100 kcps) and a color CCD camera (500 × 582 pixels) for sample positioning. The focal spot is 1.2·0.1 mm^2^, with a 0.2 mm lateral resolution and a 100 μm beryllium window.

The Spectra ARTAX software was used for hardware control and analytical data evaluation (version 5.3.14.0, license of Bruker AXS Microanalysis GmbH, Berlin, Germany). For each acquired spectrum, the percentage ratio of the peak intensities (counts) of each element to the sum of the peak intensities of all the elements was calculated. Data are plotted as counts versus energy (keV).

The following elements were examined: Cu (line: Kα1 8.0463 keV), Pb (line: Lα1 10.551 keV), Sn (line: Kα2 25.044 keV), Fe (line: Kα1 6.4052 keV), Ca (line: Kα2 3.6888 keV), Zn (line: Kα2 8.6141 keV), Ag (line: Kα2 21.99 keV), Ti (line: Kα2 4.5058 keV), and Mn (line: Kα1 5.9003 keV).

#### 3.2.3. ICP-AES Quantitative Measurements

After the collection in the archaeological site of the find, soil samples were transferred to the laboratory, where they were dried for two days until a constant weight. Each dried soil sample was powdered and homogenized in an agate mortar and then an aliquot was digested in a closed microwave system (Multiwave PRO, Anton Paar GmbH, Graz, Austria) following the method EPA 3052 [26]; that is, 250 mg of homogenized dried samples was transferred to PTFE vessels and mineralized using two heating steps. In the first step, nitric acid, hydrochloric acid, hydrofluoric acid, and hydrogen peroxide were used. In the second step, a solution of boric acid (6%) was added to buffer the excess HF. After mineralization, the solutions were diluted to a volume of 25 mL by adding Milli-Q water and stored at 4 °C until analysis. 

Solutions were then analyzed with an ICP-AES spectrometer (Optima 8000, PerkinElmer with S10 autosampler, Waltham, MA, USA). The analyses were conducted using a calibration curve obtained by dilution (range: 0–100 mg/L) of certified standard solutions (1000 mg/L) for ICP analyses. Blanks, laboratory-fortified blanks, and laboratory-fortified samples were analyzed before and after sample solutions in order to evaluate the procedure accuracy. The limit of detection (LOD) in the solution sample at the working wavelength for each element was as follows: 0.025 mg/L for Ca at 317.933 nm, 0.050 mg/L for Fe at 238.204 nm, 0.025 mg/L for Al at 396.153 nm, 0.025 mg/L for Mg at 285.213 nm, 0.025 mg/L for K at 766.490 nm, 0.025 mg/L for Ti at 334.940 nm, and 0.025 mg/L for Mn at 257.610 nm.

The repeatability of the measurements expressed as relative standard deviation (RSD%) was always lower than 5%. The concentrations of all other measured elements (Pb, As, Cu, Cd, Zn, Cr, V, Co, and Ni) are always low in sampled soil (lower than 0.01%) and not significant.

#### 3.2.4. FTIR-ATR Spectra

The infrared analysis of coin surfaces was carried out by means of a Perkin-Elmer Spectrum 100 FT-IR spectrophotometer equipped with a universal attenuated total reflectance (ATR) sampling accessory (ZnSe cell) with a diamond window (about 2 mm in diameter). Spectral analyses were performed at a resolution of 4 cm^−1^ in the range of 4000–550 cm^−1^, scanned eight times, at room temperature and humidity. The number of scans was chosen to maximize the signal-to-noise ratio. 

#### 3.2.5. SEM-EDS

Morphological analyses and microanalyses were performed using SEM (scanning electron microscope) Quanta250 (FEI, Hillsboro, OR, USA.), in high vacuum, in secondary electron mode. The working distance was adjusted in order to obtain a suitable magnification and the accelerating voltage was 30 kV. In order to detail the state of eventual patinas or encrustations, microanalysis was performed in full-frame acquisition by EDS (energy-dispersive spectroscopy) using an Apollo X EDAX probe (EDAX, Mawah, NJ, USA) coupled with SEM.

### 3.3. Data Processing

Data analysis was performed with Excel for Windows, release 2016, and Stata Software, version 11.0 (StataCorp LP, College Station, TX, USA).

## 4. Conclusions

It is important to remember that, at the time of coins’ delivery, the analyst was not given any information regarding the origin of the specimens, the issue period, or the composition of the alloy, nor was any reference made to the hypotheses based on historical-numismatic considerations.

The non-destructive analyses carried out allowed us, despite the scarcity of complementary information, to achieve the goal of assigning the six coins to the two groups in the correct way as requested by the public institution. First, two elements were found only on the surface of two coins, that is, Ti and Mn, that were also found in soil samples appositely picked up in the archaeological area. Chemical and morphological outcomes allowed us to subdivide the six target coins into two groups that were well characterized and, therefore, well distinguishable from each other. This grouping corresponds to the set of coins from excavation (coins found in the subsoil) and to the set coming from open-air finds (coins found in the top layer of the soil). The chemical composition allows to characterize the six target coins delivered to the study with respect to the following: (i) the finding conditions: two coins were buried (566 and 568), found encrusted with earth and with oxidation patinas, while four coins, found clean, with oxidation patinas, existed mainly in the open-air (in the top soil) over centuries (542, 453, 488, and 467); and (ii) the marked chemical heterogeneity of the alloys—mostly the wide difference in copper percentage—indicates a multiplicity of provenances (and, therefore, of raw materials and workmanship) of bronze coins found in this rather popular votive site.

The archaeological site located on a mountain pass—evidence has been found to support the hypothesis of the votive character of the place—probably welcomed transhumant shepherds and/or migrants passing of different origins, and thus with different currencies as well as different travel aims and destinations. People with different origins, itineraries, and travel purposes bring coins of different composition according to their origins, rather than collected in trades during travel.

The results indicate a wide chemical heterogeneity of these finds as well as a different history and modality of findings. It is likely that the minor elements found in the specimens analyzed reveal that different raw minerals, or melted again alloys (like old coins that have gone out of circulation), were used to manufacture the various coins.

Specimens of the group containing four coins are devoid of characteristics of coins exposed to subsoil for long periods of time; this could suggest a different provenance. 

In conclusion, this study demonstrated the possibility of attributing a small subset of coins to the two groups based on their chemical and morphological characteristics, using only non-destructive methods. The findings support numismatists who were not convinced about considering all coins to come from the same site on the basis of archaeological documentations. For this reason, they will not consider all of the coins to have interpretive hypotheses, but only those coming from archaeological excavations. In the future, the public institution will decide whether to continue the analyses on a greater number of coins to reinforce the information acquired.

## Figures and Tables

**Table 1:** µ-XRF signals (counts) of selected elements (average of versus and recto analysis) and Pb/Cu and Sn/Cu ratios obtained for all six coins analyzed.

Coin Number	Counts	Cu	Pb	Sn	Ca	Fe	Ag	Ti	Mn	Zn	Pb/Cu	Sn/Cu
**542**	**Average**	**228,719**	**92,501**	**25,335**	**5523**	**2307**	<100	<20	<100	<100	**0.41**	**0.11**
	SD	11,079	12,348	2132	441	482	*	*	*	*	0.07	0.01
**568**	**Average**	**518,505**	**17,656**	**17,602**	**2012**	**16,793**	**23,636**	**199**	**585**	<100	**0.04**	**0.04**
	SD	202,308	4266	2216	1123	4628	6189	228	152	*	0.02	0.02
**566**	**Average**	**492,103**	**95,768**	**1362**	**2285**	**9918**	<100	**72**	**310**	<100	**0.21**	**0.003**
	SD	115,653	41,448	442	1353	8146	*	67	249	*	0.14	0.001
**453**	**Average**	**1,086,044**	**6004**	**2113**	**960**	**2318**	<100	<20	<100	<100	**0.006**	**0.002**
	SD	44,968	2332	194	563	305	*	*	*	*	0.002	0.000
**488**	**Average**	**1,083,968**	**869**	**664**	**424**	**12,607**	<100	<20	**323**	<100	**0.001**	**0.001**
	SD	92,111	449	161	324	7318	*	*	318	*	0.000	0.000
**467**	**Average**	**882,254**	**10,367**	**2837**	**2197**	**25,950**	<100	<20	<100	**59,741**	**0.014**	**0.004**
	SD	197,141	6387	1376	1157	12,715	*	*	*	15,645	0.011	0.003
**soil**	**Average**	**206**	**239**	<100	**906**	**78,768**	<100	**1520**	**2489**	**851**	*	*
	SD	51	95	*	61	11,639	*	187	309	48	*	*

* Not determinable.

**Table 2:** Peak area vs. total peak area (in %) of the coins obtained by the µ-XRF technique expressed as the average, standard deviation, and percentage relative standard deviation for the nine elements detected.

Coin #	Peak Area vs. Total Peaks Area (%)	Cu	Pb	Sn	Ca	Fe	Ag	Ti	Mn	Zn
**542**	**average**	**64.5**	**26.0**	**7.14**	**1.56**	**0.65**	**<0.03**	**0.006**	**<0.03**	**<0.03**
	SD	2.8	3.0	0.42	0.14	0.12	*	0.000	*	*
	RSD%	4.4	11	5.8	8.9	18		4.4		
**568**	**average**	**85.1**	**3.3**	**3.24**	**0.39**	**3.29**	**4.6**	**0.049**	**0.113**	**<0.03**
	SD	7.1	1.3	1.18	0.27	2.00	2.7	0.060	0.070	*
	RSD%	8.4	39	37	68	61	58	123	62	
**566**	**average**	**81.0**	**16.5**	**0.23**	**0.40**	**1.69**	**<0.03**	**0.013**	**0.056**	**<0.03**
	SD	7.8	8.7	0.09	0.26	1.34	*	0.010	0.037	*
	RSD%	9.7	52	37	65	79		78	66	
**453**	**average**	**98.9**	**0.6**	**0.19**	**0.09**	**0.21**	**<0.03**	**<0.006**	**<0.03**	**<0.03**
	SD	0.3	0.2	0.02	0.05	0.03	*	*	*	*
	RSD%	0.3	42	9.5	62	12				
**488**	**average**	**98.6**	**0.1**	**0.06**	**0.04**	**1.18**	**<0.03**	**<0.006**	**0.033**	**<0.03**
	SD	0.8	0.0	0.02	0.03	0.73	*	*	0.029	*
	RSD%	0.8	48	28	80	62			90	
**467**	**average**	**89.3**	**1.2**	**0.32**	**0.25**	**2.92**	**<0.03**	**<0.006**	**<0.03**	**6.01**
	SD	2.9	0.9	0.21	0.17	1.95	*	*	*	0.43
	RSD%	3.2	79	67	70	67				7.1

* Not determinable.

**Table 3:** Elemental composition of the soil sampled in the archaeological site obtained by the ICP-AES technique (% dw = percentage calculated on the dry weight of the sample).

	Ca	Fe	Al	Mg	K	Ti	Mn
average (% dw)	0.228	2.743	3.49	0.229	0.660	0.235	0.119
SD	0.016	0.067	0.46	0.070	0.055	0.003	0.003

## Data Availability

The data presented in this study are available upon request from the corresponding author.

## Sample Availability

Samples of the compounds are not available from the authors. The analyzed coins are located at the Museum of Natural History and Archaeology in Montebelluna (Treviso, Italy).
